# Outcomes, Predictors of Retreatment, and Complications After Repeat Gamma Knife Radiosurgery for Trigeminal Neuralgia: A Single-Center Retrospective Cohort Study

**DOI:** 10.7759/cureus.89485

**Published:** 2025-08-06

**Authors:** Biljana Seha, Vojislav Bogosavljevic, Danica Grujicic, Vuk Djulejic, Mihailo Milićević, Rosanda Ilic, Koca Cicarevic, Marija Jovanovic, Slobodan Kapor

**Affiliations:** 1 Department of Neurosurgery, University Clinical Centre of Serbia, Belgrade, SRB; 2 Department of Neurosurgery, University of Belgrade - Faculty of Medicine, Belgrade, SRB; 3 Department of Anatomy, University of Belgrade - Faculty of Medicine, Belgrade, SRB; 4 Department of Radiology, University Clinical Centre of Serbia, Belgrade, SRB; 5 Department of Neuroanatomy, Institute of Anatomy Niko Miljanic, University of Belgrade - Faculty of Medicine, Belgrade, SRB

**Keywords:** gks, neuro radiology, stereotactic and functional neurosurgery, trigeminal nerve, trigeminal neuralgia

## Abstract

Objective: This study was conducted to evaluate the outcomes of repeat Gamma Knife surgery (GKS) in patients with recurrent trigeminal neuralgia (TN), identify predictors for retreatment, and characterize associated complications.

Methods: Among 198 patients initially treated with GKS for TN, 34 (17.2%) underwent a second procedure due to recurrent or persistent pain. After applying the eligibility criteria, 25 patients were analyzed. Factors associated with retreatment were assessed, and complications and pain relief outcomes following repeat GKS were evaluated.

Results: Prior microvascular decompression (MVD) and lower initial radiation dose were significantly associated with need for retreatment (p < 0.001 and p = 0.014, respectively). Neurovascular conflict and prior rhizotomy were not statistically significant predictors. Facial hypoesthesia was the most common complication after repeat GKS (64%). Repeat treatment yielded moderate pain relief in most patients, but durable long-term benefit was limited, consistent with literature reporting 40-60% efficacy.

Conclusions: Repeat GKS is a valuable option for recurrent TN but presents a risk of sensory complications. Prior MVD and lower initial dose are important predictors of retreatment necessity, guiding individualized patient counseling and treatment planning.

## Introduction

Trigeminal neuralgia (TN) is a long-lasting and often debilitating pain condition that mainly affects the sensory areas of the trigeminal nerve, the largest cranial nerve (cranial nerve V). The trigeminal nerve carries facial sensations and helps control chewing. It has three main branches: the ophthalmic (V1), maxillary (V2), and mandibular (V3) divisions. These branches come together at the trigeminal, or Gasserian, ganglion and then move into the brainstem at the level of the pons [1]. Classical TN usually results from neurovascular compression where the trigeminal nerve enters the brain, causing damage to the protective myelin. This damage leads to abnormal nerve signals, resulting in the sharp, electric shock-like facial pain episodes that patients experience [2,3]. TN falls into two main categories: primary (or classical) TN and secondary TN. Primary TN occurs without a known cause or is linked to vascular compression. Secondary TN develops from other health issues, such as multiple sclerosis, brain tumors, or injuries [4].

The condition significantly lowers quality of life, with pain episodes that can come on suddenly or be triggered by everyday activities such as talking, eating, brushing teeth, or being around cold air. These pain episodes not only impact physical ability but also cause substantial emotional distress, anxiety, and depression in those affected [5]. Given the debilitating nature of TN, prompt and effective treatment is essential. The first-line approach usually includes medication, with carbamazepine recognized as the best option. Other anticonvulsants like oxcarbazepine and gabapentin are also frequently used [6]. Although many patients initially respond well to these medications, a significant number eventually do not respond to treatment or suffer from intolerable side effects such as dizziness, drowsiness, liver issues, or cognitive problems [7,8]. For those who do not respond to medication, surgical and radiosurgical options may be necessary. Among these, Gamma Knife radiosurgery (GKS) has become popular due to its minimally invasive approach and good safety record. GKS is a precise technique that delivers a strong dose of targeted radiation to a specific area, usually the cisternal segment or the retrogasserian part of the trigeminal nerve, without needing surgery [9]. This method is believed to work by damaging the nerve fibers and stopping pain signals from being transmitted through the affected area [10]. After GKS, about 70-85% of patients report pain relief, which often occurs within weeks or months of treatment [11].

However, follow-up studies indicate that it is common for pain to return after some time. Research shows that many patients experience a recurrence of pain within a few years, leading to the need for further treatment [12]. Repeat GKS has been successful in many instances, but comes with a higher risk of side effects. The most common complication after repeat treatment is facial numbness, with other issues including changes in taste, dry eyes, and, in rare cases, painful sensations [13-15]. Investigating the reasons for treatment failure or pain recurrence after initial GKS is ongoing. Some important factors that influence these outcomes include receiving a lower radiation dose (e.g., less than 80 Gy), previous surgeries like microvascular decompression (MVD), having a neurovascular conflict, and anatomical differences in nerve structure [16,17]. Factors related to patients, such as age, gender, duration of pain, and which trigeminal branches are affected, may also affect results [18]. These complexities make managing recurrent TN after initial GKS challenging. Current strategies for retreatment are not yet standardized, and the choice to pursue a second GKS must consider the chance of pain relief against possible new complications or worsening of existing ones.

Therefore, thorough research into what influences both successful outcomes and side effects after repeat GKS is crucial for improving treatment approaches. This study aims to evaluate factors associated with the need for repeat GKS in patients with recurrent TN. Also, the study analyzes outcomes and complications following repeat GKS, identifying predictors of treatment response as well as side effects. By examining a uniform, single-institution cohort, this study seeks to contribute to evidence-based decision-making in the management of recurrent TN.

Several large series and systematic reviews have evaluated outcomes of repeat GKS for TN, but many are limited by heterogeneous treatment protocols or a lack of detailed complication stratification. This study provides a focused analysis from a single high-volume center with uniform treatment protocols. Furthermore, we identify and quantify the impact of prior microvascular decompression and initial dose as predictors of retreatment, providing practical guidance for patient selection and making treatment decisions. Our study offers a unique contribution within the context of a Southeastern European patient population, an area underrepresented in current literature.

## Materials and methods

This is a retrospective cohort study conducted at the Neurooncology Center, University Clinical Center of Serbia, Belgrade, Serbia. The study was approved by the Ethics Committee of the University of Belgrade - Faculty of Medicine (approval number: 9700/12-БШ, dated December 22, 2020). All participants provided written informed consent for both treatment and the use of anonymized data in research.

Eligibility criteria

Inclusion criteria for patient selection were: adults diagnosed with classical TN, confirmed by clinical evaluation and MRI, medically refractory TN, defined as inadequate pain relief with at least two standard pharmacological treatments, who underwent initial GKS at our center between November 2015 and June 2019, with a minimum of 12 months of follow-up after initial GKS. Patients with bilateral TN, secondary TN due to other causes (e.g., tumor, multiple sclerosis, trauma), and incomplete clinical or radiological records were excluded.

Of 198 patients initially treated, 34 patients required repeat GKS for recurrent or persistent pain. After applying exclusion criteria, 25 patients were included in the final analysis of repeat GKS outcomes.

GKS technique

All patients underwent GKS at the Neurooncology Center, University Clinical Center of Serbia, using the Leksell Gamma Knife Perfexion system (Elekta AB, Stockholm, Sweden). For target visualization, high-resolution MRI was used with constructive interference in steady-state (CISS) and gadolinium-enhanced T1-weighted sequences. Treatment planning was conducted using Leksell GammaPlan® software (Elekta AB), and all patients were treated under local anesthesia and stereotactic Leksell frame fixation. Based on trigeminal nerve length and anatomy, the target was located in the cisternal segment or retrogasserian portion. For the initial GKS, marginal doses ranged from 70 Gy to 90 Gy, with most patients receiving 80-85 Gy at the 50% isodose line.

For the second GKS, the prescribed dose was typically 70-80 Gy, adjusted based on anatomical proximity to the brainstem, nerve length, and prior dose. There was no strict dose standardization, but the dosing strategy followed internal institutional protocols, considering safety margins and prior dose exposure. Beam collimation was performed using a 4-mm collimator, with shots placed along the trigeminal nerve segment to cover the target volume while minimizing radiation to adjacent structures. All procedures were performed by the same radiosurgery team to ensure consistency in planning and delivery.

**Figure 1:**
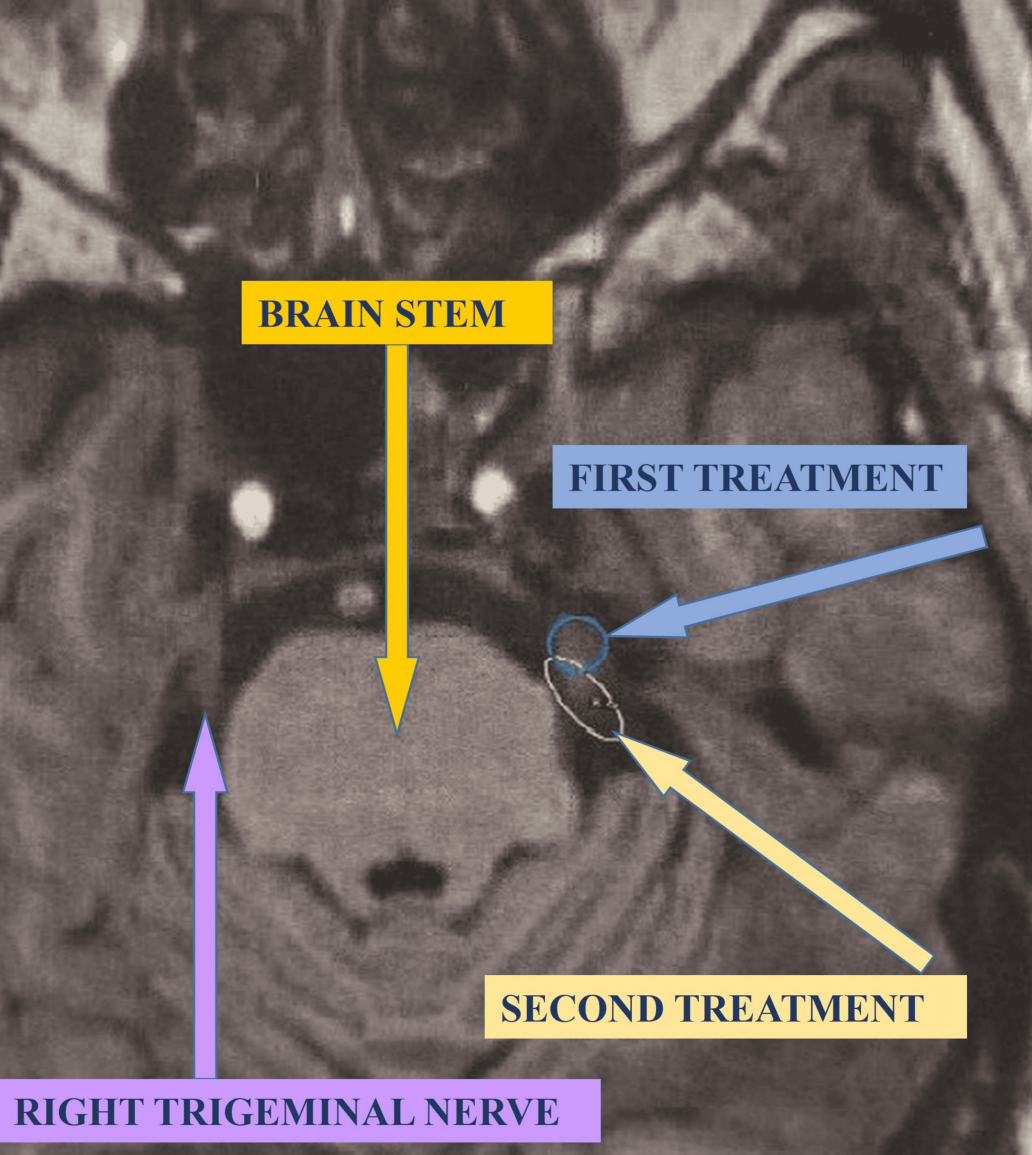
Gamma Knife surgery treatment and retreatment for left sided trigeminal neuralgia

Figure 1 shows the two GKS treatments for left-sided TN.

Data collection

Clinical and demographic data were extracted from patient records, including age, gender, pain laterality, previous surgical history (e.g., MVD or rhizotomy), presence of neurovascular conflict (NVC), radiation dose, and target site. Patients underwent standardized follow-up at one, three, six, and 12 months post GKS, then annually. Follow-up included in-person neurological evaluations when possible, or structured telephone interviews when in-person was not feasible, and MRI scans as clinically indicated to assess for structural changes or complications. Outcome measures included pain relief based on the Marseille Pain Scale, a five-grade system commonly used in European neurosurgical practice, and complication rates such as hypoesthesia, dysgeusia, dry eye, and difficulty in mastication.

Data analysis

Statistical analysis was performed using SPSS Statistics for Windows, version 11.0 (Released 2002; SPSS Inc., Chicago, Illinois, United States). Descriptive statistics summarized baseline characteristics. Chi-square and Fisher’s exact tests were used for categorical variables, while independent samples t-tests evaluated continuous variables. Logistic regression was applied to identify independent predictors of repeat GKS. A p-value of ≤ 0.05 was considered statistically significant. 

## Results

Of the initial 198 patients treated with GKS for TN, 34 (17.2%) required a second procedure due to persistent or recurrent pain. After exclusions, 25 patients formed the repeat treatment cohort, composed of 18 females and seven males, with a mean age of 68 years (range: 44-84) and an average symptom duration of 6.3 years prior to the initial GKS.

Following repeat GKS, 72% (n=18) achieved partial to complete pain relief (Marseille grades I-III), and 44% (n=11) reported complete or near-complete relief (grades I-II). Seven patients (28%) experienced minimal or no improvement (grades IV-V). The median time to onset of relief was 6.2 weeks (range: 3-14 weeks), and the median duration of sustained relief was 19 months (range: 6-39 months), with a median follow-up of 26 months.

Statistical analysis (Table 1) identified prior MVD as a strong predictor for retreatment (χ² = 13.72, p < 0.001, Chi-square test), and initial doses below 80 Gy were also significantly associated with recurrence (χ² = 6.03, p = 0.014, Chi-square test). A borderline association was noted for prior rhizotomy (χ² = 2.89, p = 0.089, Chi-square test), while neurovascular conflict (χ² = 3.34, p = 0.068, Chi-square test) and pain laterality or target location (not shown) did not show significant predictive value.

**Table 1:** Treatment factors and their association with retreatment need

Factor	Test Statistic	p-value	Statistically Significant	Test Used
Prior Microvascular Decompression	χ² = 13.72	< 0.001	Yes	Chi-square test
Neurovascular Conflict	χ² = 3.34	0.068	No	Chi-square test
Lower Initial Dose (<80 Gy)	χ² = 6.03	0.014	Yes	Chi-square test
Prior Rhizotomy	χ² = 2.89	0.089	No (Borderline)	Chi-square test

**Figure 2:**
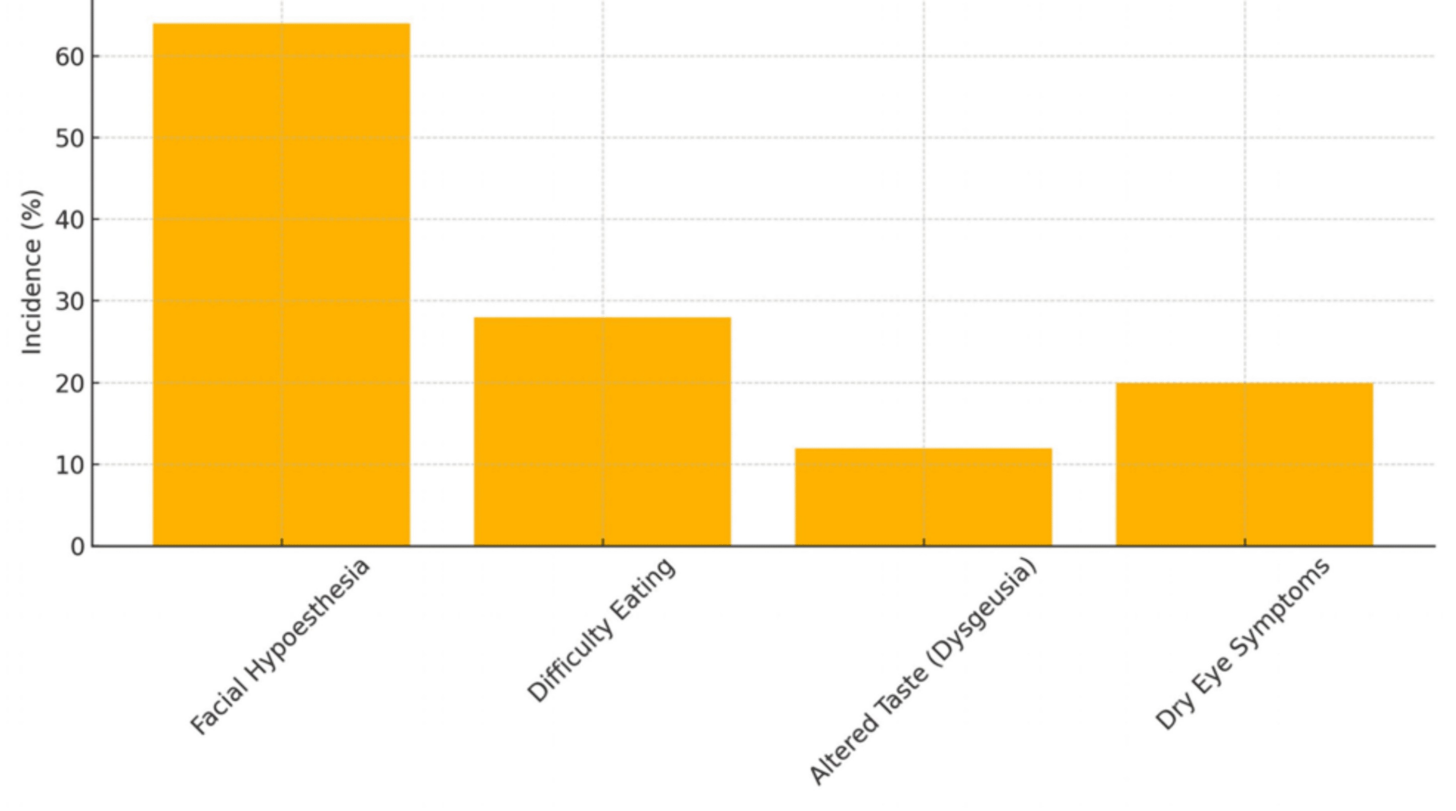
Incidence of complications after repeat Gamma Knife surgery

As summarized in Table 2, the most common postoperative complication was facial hypoesthesia, reported in 64% of patients (16 out of 25). Other notable complications included difficulty eating (28%), dry eye (20%), and dysgeusia (12%). Notably, no cases of anesthesia dolorosa were reported. Figure 2 shows the incidence of complications after repeat GKS. Table 3 displays the frequency of each complication observed in the study population.

**Table 2:** Post-treatment complications after repeat Gamma Knife surgery (N=25) NOTE: Some patients experienced more than one complication following repeat GKS; therefore, the percentages do not sum to 100%.

Complication	Frequency	Percentage
Facial Hypoesthesia	16	64%
Difficulty Eating	7	28%
Dry Eye	5	20%
Dysgeusia (Altered Taste)	3	12%
Anesthesia Dolorosa	0	0%

**Table 3:** Complication frequency

Complication	Incidence (%)
Facial Hypoesthesia	64
Difficulty Eating	28
Altered Taste (Dysgeusia)	12
Dry Eye Symptoms	20

Among the 25 patients who underwent repeat GKS, 18 (72%) achieved some level of pain relief: 11 (44%) reported complete or near-complete relief (Marseille I-II), and seven (28%) had partial relief (Marseille III). The remaining seven patients (28%) experienced minimal or no benefit (Marseille IV-V). The median time to onset of pain relief was 6.2 weeks (range: 3-14). Of the initial responders, nine patients (36%) maintained sustained pain relief (Marseille I-II without recurrence) over a median follow-up period of 26 months (range: 12-49). The median duration of sustained benefit before any recurrence was 19 months (range: 6-39). These findings confirm that while repeat GKS provides moderate short-term relief for most patients, long-term durability remains limited in a significant subset. The statistical significance of various treatment factors is summarized in Table 4.

**Table 4:** Treatment factors and significance

Factor	p-value	Statistically Significant
Prior Microvascular Decompression	< 0.001	Yes
Neurovascular Conflict	0.068	No
Lower Initial Dose	0.014	Yes
Prior Rhizotomy	0.089	No (Borderline)

## Discussion

GKS is a well-established, minimally invasive, and effective treatment for TN, particularly in patients who are refractory to pharmacologic management or unsuitable for open surgical procedures [19,20]. While initial pain relief following GKS is generally high, a proportion of patients experience persistent or recurrent symptoms over time, necessitating repeat intervention [21]. Although repeat GKS can restore pain control, it is associated with a higher rate of complications, most commonly sensory disturbances such as facial numbness [22]. The present study aimed to evaluate the long-term effectiveness and safety of repeat GKS and to identify clinical factors predictive of the need for retreatment.

The mechanism by which GKS alleviates TN symptoms involves targeted delivery of high-dose ionizing radiation to the proximal trigeminal nerve root entry zone (REZ), thereby selectively damaging pain-conducting fibers while preserving surrounding structures [16,23]. Histopathological studies have demonstrated that radiosurgery induces localized demyelination, axonal degeneration, and gliosis in the affected nerve segment, without significant necrosis or widespread structural disruption [23]. This focused approach accounts for the efficacy and low morbidity associated with initial GKS treatment. However, the effectiveness of the procedure is dose-dependent, and escalating the radiation dose, though associated with improved pain control, also increases the risk of sensory side effects, including hypoesthesia and paresthesia [24].

Our findings support the conclusion that lower initial radiation doses, specifically those under 80 Gy, are significantly associated with an increased likelihood of retreatment. This observation aligns with prior dose-response studies, which have shown that higher doses are more likely to produce durable pain relief but may also heighten the risk of sensory complications [24,25]. The need to balance therapeutic efficacy with the potential for toxicity underscores the importance of individualized dose planning based on patient-specific anatomy, symptom severity, and prior interventions.

The data also indicate that prior MVD is a strong predictor of repeat GKS, with patients who had previously undergone MVD exhibiting nearly five times greater odds of requiring another procedure. This population may represent a more refractory clinical subgroup with altered nerve morphology, persistent central sensitization, or incomplete disruption of pain pathways, all of which could diminish the success of radiosurgical treatment [26,27]. Additionally, prior surgical manipulation may lead to changes in the geometry or vascularization of the trigeminal nerve, potentially affecting how the radiation dose is delivered and absorbed [27].

Patients who underwent repeat GKS also received significantly lower mean initial doses than those who achieved long-term relief with a single procedure, further suggesting that insufficient initial dosing may fail to produce adequate disruption of pain-transmitting fibers [24]. This reinforces the importance of optimizing the initial treatment protocol to reduce the likelihood of recurrence and the need for retreatment.

The role of neurovascular conflict in predicting outcomes of GKS remains controversial. Although traditionally considered a principal cause of TN, our study found no statistically significant association between neurovascular conflict and the need for repeat GKS. This may be due to limitations in imaging techniques or the dynamic nature of vascular compression, which can vary over time [28]. These findings support the growing recognition that pain recurrence may be driven not only by mechanical factors but also by intrinsic properties of nerve excitability and central pain modulation [4].

Sensory complications remain the most common adverse event associated with repeat GKS, with facial numbness occurring in 64% of patients in our cohort. This rate exceeds that observed after primary treatment and likely reflects cumulative radiation exposure and heightened vulnerability of previously treated neural tissue [26]. While most cases of numbness are mild and tolerable, some patients report distressing dysesthesia or functional impairment. Additional side effects, including altered taste and dry eye, may arise due to radiation spread to adjacent cranial nerves or their central connections [26]. Nonetheless, given the severe pain and reduced quality of life associated with untreated TN, many patients consider these complications an acceptable trade-off for symptom relief.

The clinical implications of these findings are significant. Recognizing prior MVD and lower initial radiation dose as predictors of repeat treatment can inform shared decision-making and guide individualized treatment strategies. For patients identified as high-risk for recurrence, more aggressive initial dosing or consideration of alternative therapies such as neuromodulation or percutaneous interventions may be appropriate. Moreover, given that only 40-60% of patients achieve durable relief after a second GKS, careful patient selection and expectation management are essential [4].

Looking ahead, future research should focus on refining radiation delivery through advanced dose planning algorithms and investigating predictive biomarkers to stratify patients by recurrence risk. High-resolution imaging and computational modeling may improve targeting accuracy, while insights into the molecular and electrophysiological effects of radiation on nerve tissue could lead to novel strategies that enhance efficacy without increasing toxicity. Together, these advances could substantially improve outcomes for patients undergoing GKS for TN.

## Conclusions

In this study, we evaluated the results of repeat GKS in patients with ongoing TN and identified factors linked to the need for further treatment. Our findings show that a history of MVD and a lower initial radiation dose significantly increase the likelihood of a second GKS. These elements may indicate greater disease complexity and ineffective initial treatment, respectively. Repeat GKS provides moderate pain relief for a substantial proportion of patients with recurrent or persistent TN. However, the durability of pain control remains variable, and a notable subset of patients may experience limited long-term benefit. Careful patient selection and individualized treatment planning are essential to optimize outcomes while minimizing complications. The rate of complications, especially facial numbness, is significant and needs careful consideration alongside the potential benefits of the procedure. Doctors should factor these predictors into their treatment plans and patient discussions, emphasizing the need for individualized strategies. More research is needed to refine dosing guidelines, enhance patient selection through advanced imaging and assessments, and develop additional therapies to improve long-term outcomes. Ultimately, repeat GKS remains a valuable option for managing recurring TN, but attention to past surgeries and initial treatment parameters is crucial for maximizing effectiveness and minimizing side effects.
